# Topical Application of Glycolipids from *Isochrysis galbana* Prevents Epidermal Hyperplasia in Mice

**DOI:** 10.3390/md16010002

**Published:** 2017-12-25

**Authors:** Azahara Rodríguez-Luna, Elena Talero, María del Carmen Terencio, María Luisa González-Rodríguez, Antonio M. Rabasco, Carolina de los Reyes, Virginia Motilva, Javier Ávila-Román

**Affiliations:** 1 Department of Pharmacology, Faculty of Pharmacy, Universidad de Sevilla, 41012 Sevilla, Spain; arodriguez53@us.es (A.R.-L.); etalero@us.es (E.T.); motilva@us.es (V.M.); 2 Department of Pharmacology, Faculty of Pharmacy, University of Valencia, 46010 Valencia, Spain; carmen.terencio@uv.es; 3 Institute of Molecular Recognition and Technological Development (IDM), 46100 Valencia, Spain; 4 Department of Pharmaceutical Technology, Faculty of Pharmacy, Universidad de Sevilla, 41012 Sevilla, Spain; malugoro@us.es (M.L.G.-R.); amra@us.es (A.M.R.); 5 Department of Organic Chemistry, Faculty of Marine and Environmental Sciences, University of Cadiz, 11510 Puerto Real, Cádiz, Spain; carolina.dereyes@uca.es

**Keywords:** glycolipids, MGDG, skin, inflammation, epidermal hyperplasia, microalgae, *Isochrysis galbana*

## Abstract

Chronic inflammatory skin diseases such as psoriasis have a significant impact on society. Currently, the major topical treatments have many side effects, making their continued use in patients difficult. Microalgae have emerged as a source of bio-active molecules such as glycolipids with potent anti-inflammatory properties. We aimed to investigate the effects of a glycolipid (**MGMG-A**) and a glycolipid fraction (**MGDG**) obtained from the microalga *Isochrysis galbana* on a TPA-induced epidermal hyperplasia murine model. In a first set of experiments, we examined the preventive effects of **MGMG-A** and **MGDG** dissolved in acetone on TPA-induced hyperplasia model in mice. In a second step, we performed an in vivo permeability study by using rhodamine-containing cream, ointment, or gel to determinate the formulation that preserves the skin architecture and reaches deeper. The selected formulation was assayed to ensure the stability and enhanced permeation properties of the samples in an ex vivo experiment. Finally, **MGDG**-containing cream was assessed in the hyperplasia murine model. The results showed that pre-treatment with acetone-dissolved glycolipids reduced skin edema, epidermal thickness, and pro-inflammatory cytokine production (TNF-α, IL-1β, IL-6, IL-17) in epidermal tissue. The in vivo and ex vivo permeation studies showed that the cream formulation had the best permeability profile. In the same way, **MGDG**-cream formulation showed better permeation than acetone-dissolved preparation. **MGDG**-cream application attenuated TPA-induced skin edema, improved histopathological features, and showed a reduction of the inflammatory cell infiltrate. In addition, this formulation inhibited epidermal expression of COX-2 in a similar way to dexamethasone. Our results suggest that an **MGDG**-containing cream could be an emerging therapeutic strategy for the treatment of inflammatory skin pathologies such as psoriasis.

## 1. Introduction

Inflammatory skin diseases have a significant impact on the quality of life of patients; one of them is psoriasis, considered a common immune-mediated inflammatory skin disorder. It is estimated that 2–4% of the population suffers from psoriasis [1]. Although the exact mechanism of this pathology is not completely understood, it is known that both genetic predisposition and environmental factors such as stress, infection, trauma, and use of some drugs play an important role in its etiology [2]. This disease is associated with several comorbidities as cardiovascular diseases, metabolic syndrome, and psychiatric disorders.

Accumulating evidence has demonstrated that exposure of skin to the protein kinase *C* activator 12-*O*-tetradecanoylphorbol-13-acetate (TPA) induces a pleiotropic tissue response and promotes macroscopic lesions, peeling, and erythema, mimicking an apparent psoriasis phenotype. Furthermore, an increase in epidermal thickness has been observed due to the hyperproliferation and aberrant differentiation of keratinocytes as well as the infiltration of inflammatory leukocytes into the epidermis and dermis [3]. Activated leukocytes cause uncontrolled production of reactive oxygen species (ROS), leading to peroxidative damage to skin membranes and contributing to the exacerbation of lesions. Moreover, these immune cells release growth factors, chemokines, and pro-inflammatory cytokines such as tumor necrosis factor (TNF)-α, interleukin (IL)-6, IL-1β and IL-17, which interact as a network in the pathogenesis of psoriasis [4]. The inducible enzyme cyclooxygenase-2 (COX-2) has also been demonstrated to play a pivotal role in skin proliferative disorders through overproduction of pro-inflammatory prostaglandins such as PGE_2_ [5]. Currently, the treatment of psoriasis includes topical agents (corticoids, vitamin d derivatives, retinoids, and calcineurin inhibitors), photo-chemo-therapy, and systemic treatments (immunosuppressants and biological drugs) [6]. However, many patients, especially those with moderate to severe generalized psoriasis, are not adequately treated with effective or long-term therapies and most of them have various degrees of side effects. Thus, the development of well-tolerated immune-modulatory topical agents can offer an alternative option for the treatment of psoriatic patients.

Microalgae have emerged as a source of bioactive compounds, including lipids, proteins, polysaccharides, and carotenoids, which have attracted the interest of the pharmaceutical industry based on their anti-oxidant, anti-inflammatory, or anti-carcinogenic activity in different skin inflammatory models [7]. Recently, the anti-inflammatory activity of galactosylglycerides isolated from the marine microalga *Isochrysis galbana* (*I. galbana*), including a monogalactosyldiacylglycerol (**MGDG**) fraction [8] and the pure compound monogalactosylmonoacylglyceride (2*S*)-1-*O*-[(6*Z*,9*Z*,12*Z*,15*Z*)-octadeca-6,9,12,15-tetraenoyl]-3-*O*-β-d-galactopyranosylglycerol (**MGMG-A**) (*data not shown*), has been reported through the inhibition of TNF-α production in LPS-stimulated THP-1 human macrophages. However, data on these compounds’ effects on skin inflammatory pathologies have not yet been collected.

Given the interesting anti-inflammatory properties and their high yield of these products in this microalga, we evaluated the preventive effects of the galactosylglycerides **MGMG-A** and **MGDG** from *I. galbana* in a murine model of TPA-induced epidermal hyperplasia by using a topical application of acetone-dissolved glycolipids. However, acetone application onto the skin has been reported to exhibit several drawbacks such as the amount of this organic solvent remaining in contact with the skin, spreading of the formulation and loss of sample, heterogeneity of the dose contacting with the skin, and difficulty of applying the sample [9]. It is well known that the topical application of bio-compounds requires their incorporation into a carrier that offers stability, good permeation, and sufficient time in contact with the skin. Currently, microalgae products are being used as cosmeceuticals through their incorporation in face and skin care products [10]. Therefore, our next objective was to use a pharmaceutical carrier to solve the above limitations of glycolipid solutions. The formulation of these substances involves the selection of appropriate combinations of formula ingredients with the aim of exerting a desirable local or systemic effect. Among them, topical formulations of different natures are used, including ointments, creams, and hydrophilic gels, in which the active compound is suspended or dissolved. In the present study, once a topical formulation was selected, we finally aimed to study its effect on a TPA-induced hyperplasia model and determinate its benefit to epidermal skin.

## 2. Results

### 2.1. Effects of Glycolipids on IL-6 and IL-8 Production in TNF-A-Stimulated HaCaT Human Keratinocytes

Non-cytotoxic concentrations of the monogalactosylmonoacylglyceride (2*S*)-1-*O*-[(6*Z*,9*Z*,12*Z*,15*Z*)-octadeca-6,9,12,15-tetraenoyl]-3-*O*-β-d-galactopyranosylglycerol (**MGMG-A**) and the monogalactosyldiacylglycerol fraction (**MGDG**) were selected to evaluate their effects on pro-inflammatory cytokines IL-6 and IL-8 production in HaCaT cells. The cytotoxic effect of **MGMG-A** and **MGDG** fraction was studied using the SRB method, resulting in 100% viability at the tested concentrations (Table S1).

**Figure 1:**
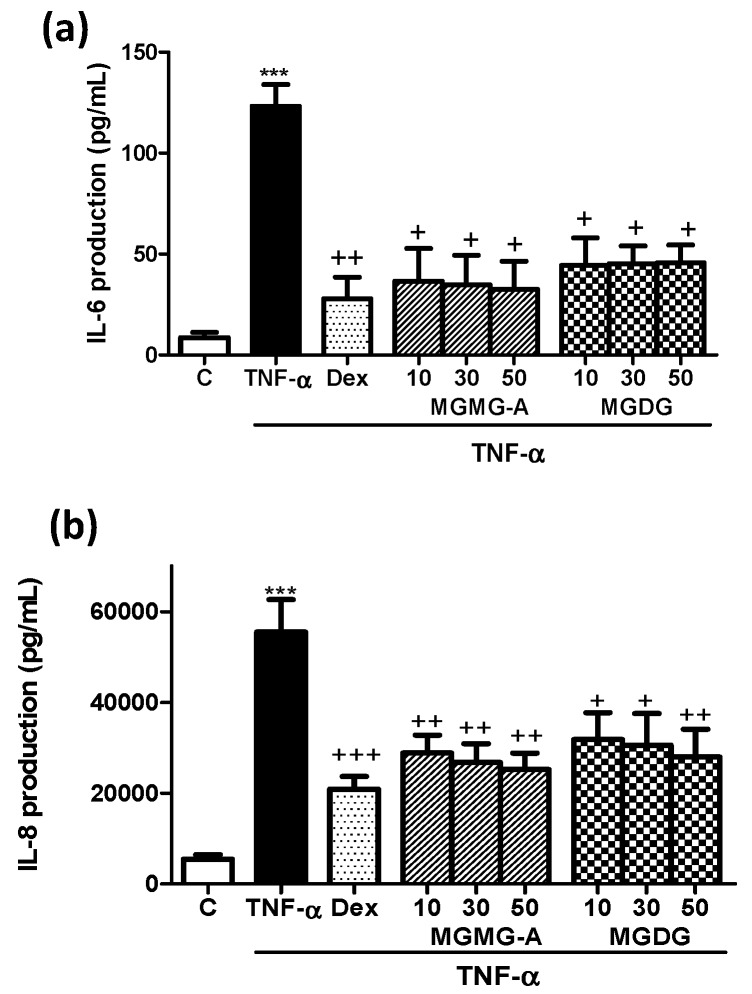
Effects of glycolipids from *I. galbana* on IL-6 and IL-8 production in TNF-α-stimulated HaCaT human keratinocytes. (**a**) IL-6 levels and (**b**) IL-8 levels in TNF-α-stimulated HaCaT human keratinocytes. Cells were pre-incubated with the glycolipid **MGMG-A** (10, 30, 50 µM) and the fraction **MGDG** (10, 30, 50 µg/mL) for 1 h, and then stimulated with TNF-α (10 ng/mL) for 24 h. Dexamethasone (Dex) was used as a positive reference compound at 1 µM. After 24 h, the production of cytokines in the supernatants was measured by ELISA assay. Results are representative of six independent experiments (*n* = 6). Values are means with standard errors represented by vertical bars. Mean value was significantly different compared with the control group (*** *p* < 0.001; Student *t* test). Mean value was significantly different compared with the TNF-α group (+ *p* < 0.05, ++ *p* < 0.01, +++ *p* < 0.001; one-way ANOVA followed by Bonferroni’s Multiple Comparison test).

TNF-α-stimulated HaCaT cells manifested high IL-6 and IL-8 levels in comparison with unstimulated control cells (*p* < 0.001) (Figure 1). Pre-treatment with the reference compound dexamethasone (Dex) as well as **MGMG-A** (10, 30, and 50 µM) and **MGDG** fraction (10, 30, and 50 µg/mL) significantly inhibited IL-6 and IL-8 production, with no significant differences between the different tested concentrations.

### 2.2. Topical Application of Acetone-Dissolved Glycolipids Inhibits Skin Inflammation and Hyperplasia in the Murine TPA-Induced Model

**Figure 2:**
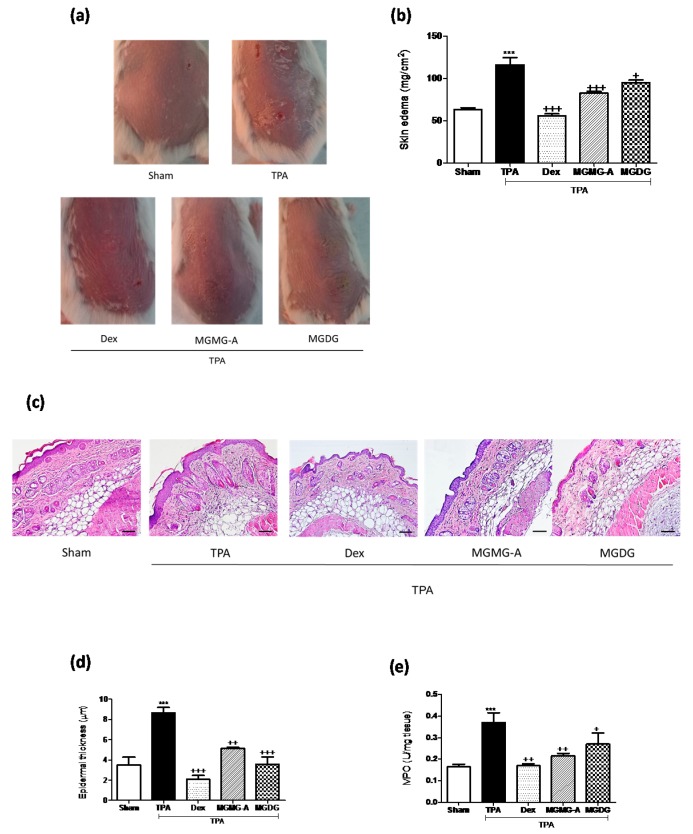
Topical application of acetone-dissolved glycolipids from *I. galbana* inhibits skin inflammation and hyperplasia on the murine 12-*O*-tetradecanoylphorbol-13-acetate (TPA)-induced model. The glycolipid **MGMG-A** (200 µM per site) or the fraction **MGDG** (200 µg/mL per site) were topically administered 30 min before TPA application (2 nmol per zone) during three consecutive days. Dex was used as a positive reference compound (200 µg per site). (**a**) Representative images of macroscopic appearance of the dorsal skin; (**b**) skin edema as punch biopsy; weight of edema (mg/cm^2^) was employed as marker of inflammatory skin process; (**c**) histological appearance of mouse dorsal skin after H&E-staining (*n* = 4); Bar = 100 µm. Original magnification 100×. (**d**) Epidermal thickness assessment in H&E-stained skin slides; (**e**) yeloperoxidase (MPO) activity in dorsal skin. Values are means with standard errors represented by vertical bars. Data are means ± SEM (*n* = 10 mice/group). Mean value was significantly different compared with the sham group (*** *p* < 0.001; Student *t* test). Mean value was significantly different compared with TPA group (+ *p* < 0.05, ++ *p* < 0.01, +++ *p* < 0.001; one-way ANOVA followed by Bonferroni’s Multiple Comparison test).

We studied the effect of **MGMG-A** and **MGDG** on the murine TPA-induced epidermal hyperplasia model, which reproduces certain biochemical and histopathological parameters typical of human psoriasis [11]. TPA administration to mouse skin resulted in the development of macroscopic lesions (Figure 2a) and skin edema, confirmed by a higher weight of the 1 cm^2^ punch biopsies compared with the sham group (*p* < 0.001) (Figure 2b). Topical treatment with Dex (200 µM), **MGMG-A** and **MGDG** (200 µM or 200 µg/mL, respectively) 30 min prior to TPA application inhibited macroscopic damage and the skin punch weight (*p* < 0.001 and *p* < 0.05, respectively), suggesting an inhibition of skin edema (Figure 2b). We next examined hematoxylin- and eosin-stained sections of mouse skin (Figure 2c). Consistent with macroscopic changes, TPA-treated animals exhibited a clear evidence of edema, epidermal hyperplasia, and massive neutrophilic infiltration compared with the sham (Figure 2c). Moreover, a marked increase in epidermal thickness was evident in the TPA group (*p* < 0.001) (Figure 2d). These results correlated with increased MPO activity, an established marker for inflammatory cell infiltration into the skin (Figure 2e). Treatment with the pure compound and glycolipid fraction markedly prevented epidermal hyperplasia (*p* < 0.01 and *p* < 0.001, respectively) (Figure 2c,d), which was associated with a reduction in MPO activity, being significant for **MGMG-A** (*p* < 0.01) (Figure 2e).

**Figure 3:**
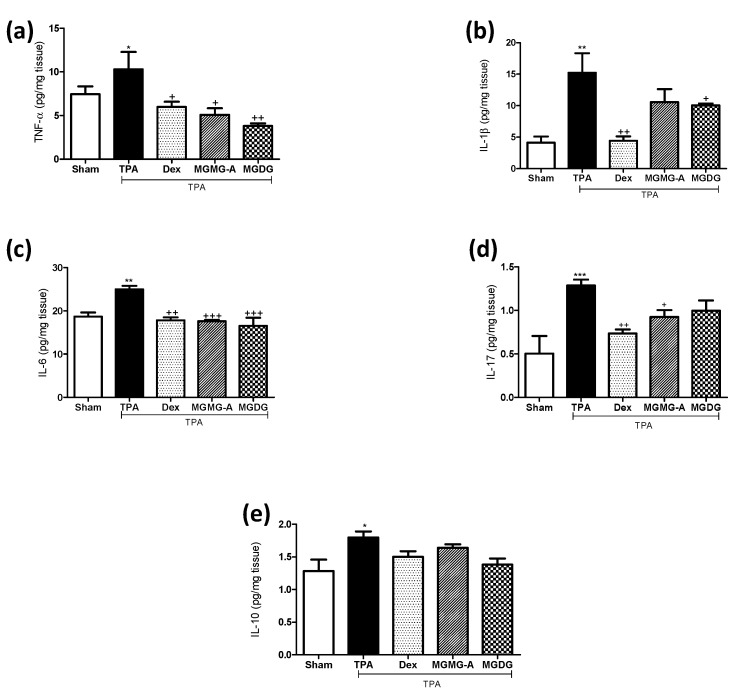
Effect of the glycolipid **MGMG-A** and the fraction **MGDG** from *I. galbana* on the production of cytokines in skin homogenates in the murine 12-*O*-tetradecanoylphorbol-13-acetate (TPA)-induced hyperplasia model. (**a**) TNF-α (pg/mg tissue); (**b**) IL-1β (pg/mg tissue); (**c**) IL-6 (pg/mg tissue); (**d**) IL-17 (pg/mg tissue); and (**e**) IL-10 (pg/mg tissue). Values are means with standard errors represented by vertical bars. Data are means ± SEM (*n* = 10). Mean value was significantly different compared with the sham group (* *p* < 0.05, ** *p* < 0.01, *** *p* < 0.001; Student *t* test). Mean value was significantly different compared with TPA group (+ *p* < 0.05, ++ *p* < 0.01, +++ *p* < 0.001; one-way ANOVA followed by Bonferroni’s Multiple Comparison test).

To support the beneficial effects of glycolipids on skin inflammation, we analyzed the production of several pro-inflammatory cytokines that are highly involved in psoriasis as well as the anti-inflammatory cytokine IL-10. Immune cell infiltration detected in the histological examination of the skin from TPA-treated mice correlated with increased levels of the pro-inflammatory cytokines TNF-α, IL-1β, IL-6 and IL-17, in comparison with the sham group (*p* < 0.05, *p* < 0.01, *p* < 0.01, and *p* < 0.001, respectively) (Figure 3). In accordance with the reduction of the skin edema, the production of TNF-α, IL-6, and IL-17 was significantly reduced in animals treated with the glycolipid **MGMG-A** (*p* < 0.05, *p* < 0.001, *p* < 0.05, respectively) (Figure 3a–d). Regarding the fraction **MGDG**, its application resulted in a strong significant suppression of TNF-α and IL-6 levels (*p* < 0.01, and *p* < 0.001) comparable to Dex (Figure 3a,c). IL-10 production analysis revealed increased levels in the TPA group when compared with the sham (*p* < 0.05). Nevertheless, pre-treatments showed lower IL-10 levels when compared to the TPA group, reflecting similar values to the sham (Figure 3e).

### 2.3. Effect of the Formulation

**Figure 4:**
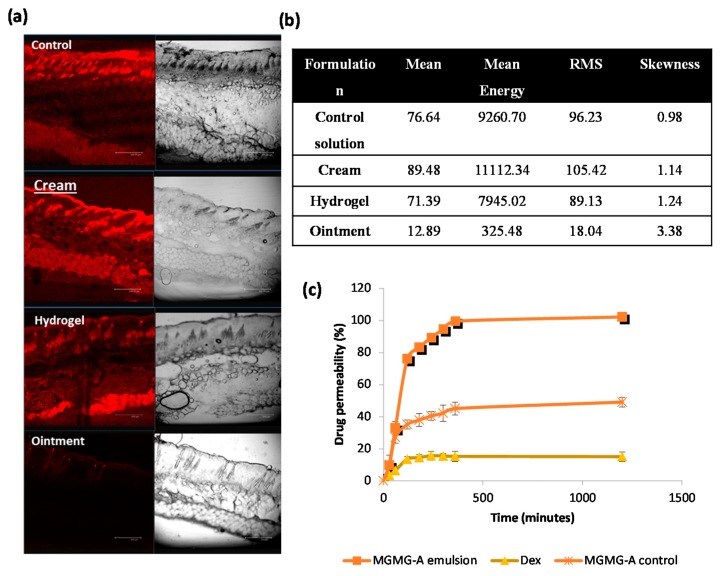
Effect of the vehicle composition and physicochemical properties of the drug on the permeation characteristics. (**a**) Confocal micrographs of mice skin cross sections corresponding to rhodamine-loaded ethanolic control solution, cream, hydrogel, and ointment. Bar = 200 µm. Original magnification 100×; (**b**) Numerical data corresponding to the intensity histogram for each sample. Mean: arithmetical mean value; mean energy: average image energy; RMS: root mean square value; skewness: skewness of the distribution; (**c**) Ex vivo permeability percentages of **MGDG** formulations in 24 h (ethanolic control solution, cream, and ointment).

The development of topical formulations implies the selection of excipients leading to improvement in the drug skin delivery. In order to evaluate the skin accumulation and penetration properties of the examined formulations, sections of the mice skin were analyzed by confocal laser scanning microscopy (CLSM) at the end of permeation experiments. For these studies, rhodamine 6G, a fluorescent hydrophobic probe, was added as a model drug [12]. The penetration depth of the fluorescent probe and the relative intensity of fluorescence in the skin layers were compared in three types of semisolid formulations (gel, cream, and ointment described in Section 4.10). Confocal images revealed that all the examined formulations penetrated deeply into the *stratum corneum* (*SC*) and diffused into the whole skin thickness, except for the ointment (Figure 4a). Cream showed the higher probe permeation in 24 h, following the control formulation containing only ethanol and incorporated into Carbopol^®^ gels. However, the rhodamine 6G incorporated into the lipid ointment was observed to show a low penetration capacity. In addition to the effect of the carrier nature, deeper skin layers were more easily visualized when ethanol was present in the composition, as occurred in all the formulations except for the ointment, where the labeling probe was dissolved in propylene glycol.

The quantitative parameters of histogram distribution revealed a higher fluorescent intensity and accumulation of rhodamine 6G in the presence of ethanol (Figure 4b). Among all the samples, the cream system offered the higher fluorescence intensity and adequate symmetry of the normal distribution of histogram.

### 2.4. Ex Vivo Permeation Studies

Permeation profiles of **MGDG** from the ethanol solution and cream through mice skin membranes were obtained from the equation described in Section 4.11. Dex-loaded cream was used as the control formulation. Results showed that permeation of **MGDG** from the cream (100 ± 1.9% of the applied dose) was twice that observed from the ethanolic control solution (49.3 ± 3.5%). On the other hand, the permeated amount of Dex from cream was lower (15 ± 3.1%) in comparison with the other preparations (Figure 4c). This value can be attributed to the lower partition coefficient of this molecule (logP 1.83) compared to **MGDG**, whose lipophilicity resembled a reference diacylglycerol in terms of lipophilic acyl groups (logP 3.85) [13]. It is well known that the partition coefficient has been widely used as a measurement for defining the lipophilicity of a drug and the diffusion efficiency across the membranes [14].

### 2.5. Topical Pre-Treatment with **MGDG**-Cream Decreases Skin Inflammation and Hyperplasia in the Murine TPA-Induced Model

**Figure 5:**
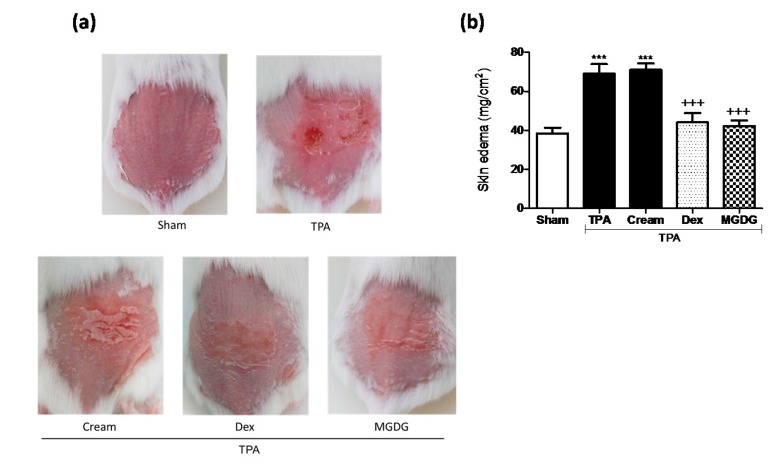

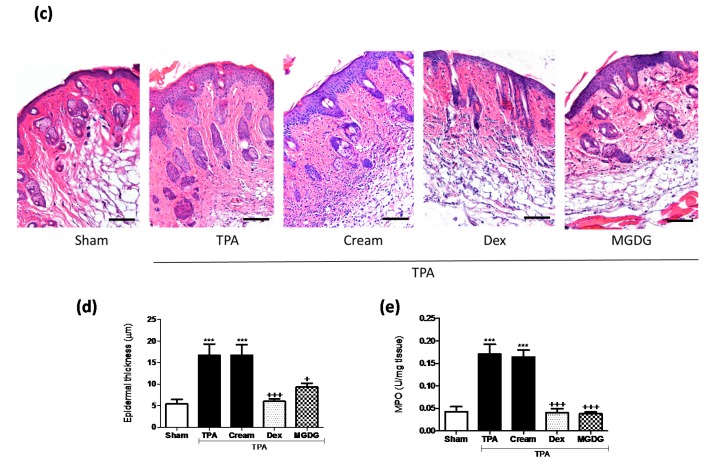
Topical pre-treatment with cream containing the glycolipid fraction **MGDG** from *I. galbana* decreases skin inflammation and hyperplasia on the murine 12-*O*-tetradecanoylphorbol-13-acetate (TPA)-induced model. Glycolipid cream formulation (100 mg per site containing 200 µg of **MGDG**), dexamethasone (Dex) (100 mg per site, equivalent at 200 µg of compound), or vehicle (cream with a comparable volume of ethanol) was topically administered from two days before hyperplasia induction and 30 min after each TPA application (2 nmol per zone for three consecutive days). Dex was used as the positive reference compound. (**a**) Representative images of macroscopic appearance of the dorsal skin; (**b**) determination of skin edema as punch biopsy weight; (**c**) histological appearance of mouse dorsal skin after H&E-staining (*n* = 4); Bar = 100 µm. Original magnification 100×. (**d**) Epidermal thickness assessment in H&E-stained skin slides; (**e**) myeloperoxidase (MPO) activity. Values are means with standard errors represented by vertical bars. Data are means ± SEM (*n* = 10 mice/group). Mean value was significantly different compared with the sham group (*** *p* < 0.001; Student *t* test). Mean value was significantly different compared with cream-TPA group (+ *p* < 0.05, +++ *p* < 0.001; one-way ANOVA followed by Bonferroni’s Multiple Comparison test).

**Figure 6:**
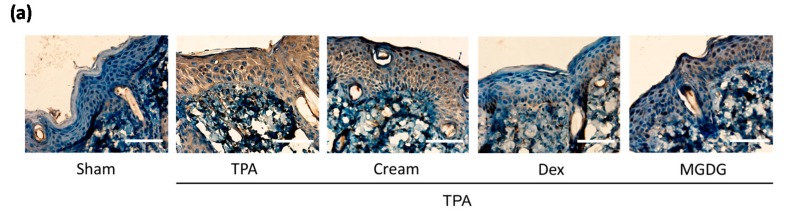

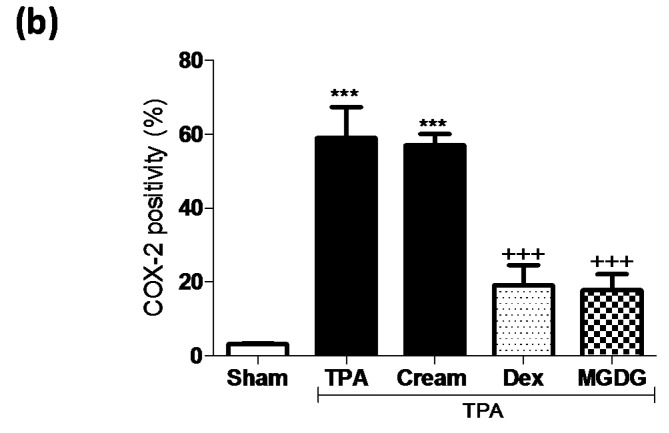
Topical pre-treatment with cream containing the glycolipid fraction **MGDG** from *I. galbana* attenuates 12-*O*-tetradecanoylphorbol-13-acetate (TPA)-induced COX-2 expression in mouse skin. (**a**) Representative photographs of epidermal COX-2 distribution by immunohistochemical detection; Bar = 200 µm. Original magnification 200×. (**b**) Positive COX-2 epidermal layer was assessed by counting the COX-2 positive cells versus total cells in different immunostained dorsal skin sections per animal. Representative photomicrographs showing positive epidermal COX-2 staining yielded a brown product. Values are means with standard errors represented by vertical bars. Data are means ± SEM (*n* = 4). Mean value was significantly different compared with the sham group (*** *p* < 0.001; Student’s *t* test). Mean value was significantly different to the cream-TPA group (+++ *p* < 0.001; one-way ANOVA followed by Bonferroni’s Multiple Comparison test).

We evaluated the effect of the **MGDG**-cream formulation on the murine TPA-induced epidermal hyperplasia model. This cream formulation enabled lipid preservation and high permeation in comparison with the acetone vehicle. After treatment with TPA for three consecutive days, mice exhibited the expected psoriasis phenotype, including peeling, erythema, and thickening of the back skin, accompanied by a marked increase in dorsal skin thickness, weight, and substantial inflammatory cell infiltration in the dermis (*p* < 0.001) (Figure 5). Pre-treatment with **MGDG**-cream (100 mg per site containing 200 µg of **MGDG**) attenuated the macroscopic lesions formation (Figure 5a) and significantly reduced skin edema (*p* < 0.001) when compared with the cream-TPA group (Figure 5b). These results were accompanied by a clear inhibition of MPO activity following **MGDG**-cream administration (*p* < 0.001); interestingly, the glycolipid formulation was as effective as the reference topical treatment with Dex-cream, reaching similar levels to those in the healthy group (Figure 4e). Histological analysis of H&E-stained skin lesions confirmed an improvement in the microscopic features of hyperplasia in mice treated with **MGDG**-cream, evidenced by a reduction of epidermal thickness (*p* < 0.05) in relation to the cream-TPA group (Figure 5c,d). It is known that COX-2 plays an important role in skin pathologies. Immunohistochemical analysis of this enzyme showed that stimulation with TPA significantly increased COX-2-positive cell numbers (*p* < 0.001), predominantly localized in the epidermal layer (Figure 6a), when compared with the sham group. As shown in Figure 6b, skin from **MGDG**-cream-treated mice revealed a significant downregulation in the number of epidermal COX-2-positive stained cells in comparison with the cream-TPA group (*p* < 0.001).

## 3. Discussion

Inflammatory skin diseases have a significant impact on society, with atopic dermatitis, acne, sunburn, and psoriasis being the most common manifestations. Psoriasis is a chronic, autoimmune, and multisystem inflammatory disease that affects 2–4% of the population [15]. Currently, conventional treatments for this disease are based on the degree of severity and range from topical therapy and systemic agents through to phototherapy or combinations of those. However, many of these therapies are not recommended for the vast majority of patients afflicted with mild forms of psoriasis due to their potential risk [16]. Therefore, other treatment approaches for mild psoriasis that require topical therapy only are still needed. In this regard, natural products provide some options for increasing the safety and efficacy in the management of this pathology [17]. Microalgae species are a promising source of a variety of bioactive molecules, including polar lipids such as glycolipids. Lipid-enriched extracts or pure glycolipids have previously demonstrated their in vitro anti-inflammatory [18,19] and antitumor properties [20], which make them suitable candidates for further investigation. However, the use of galactosylglycerides to prevent skin pathologies such as psoriasis has not been previously evidenced. In this sense, we have recently observed that this kind of metabolite protects human HaCaT keratinocytes against UVB radiation through inhibition of ROS generation and a decrease in the production of the pro-inflammatory cytokine IL-6 (*data not shown*). These findings suggest that this type of molecule could play a main role not only in protecting the skin from UVB exposure but also in preventing the skin inflammatory process. In this context, we aimed to evaluate the anti-inflammatory effects of the glycolipid **MGMG-A** and **MGDG** fraction in an experimental TPA-induced hyperplasia model in mice. Moreover, we used different semisolid formulations in which the glycolipid was loaded in order to facilitate its topical application and to enhance the permeation mechanism compared to conventional liquid preparations.

Firstly, we tried to demonstrate the anti-inflammatory potential of the compounds under study in the in vitro model of TNF-α-stimulated HaCaT keratinocytes. This cytokine plays a crucial role in the pathogenesis of skin inflammatory diseases such as psoriasis [21]. We observed that pre-treatment with the compound **MGMG-A** or the fraction **MGDG** significantly reduced the production of the pro-inflammatory cytokines IL-6 and IL-8 in stimulated HaCaT keratinocytes. These results encouraged us to evaluate the preventive effects of these products on TPA-induced hyperplasia in murine skin using two experimental approaches. In the first model, glycolipids dissolved in acetone were topically administered to dorsal skin 30 min before TPA administration. Our data showed that TPA clearly caused peeling, erythema, and a strong inflammatory reaction produced by a marked influx of mononuclear and polymorphonuclear leukocytes in epidermis and dermis. Treatment of mice with the compound **MGMG-A** or the fraction **MGDG** from *I. galbana* reduced the hyperplasia manifestations and the inflammation grade, presumably due to an inhibition of inflammatory cells infiltration, as revealed by an MPO study. These findings are interesting since the cellular infiltrate has a pathogenic role in psoriasis and its control is extremely important for the attenuation of this disease [22]. Microscopic analysis of the dorsal skin was in accordance with a macroscopic study reflecting the attenuation of keratinocytes hyperproliferation in the epidermis after treatment with acetone-dissolved glycolipids. Our results are in line with a previous report that showed that topical pre-treatment with the glycoglycerolipids **MGDG**, **DGDG**, or **SQDG**, obtained from a blue-green alga, reduced inflammation in croton-oil-induced ear edema and in carrageenan-induced paw edema models [23].

The role of TNF-α, IL-17 and IL-1β in psoriasis pathogenesis has been well documented [24], being a particularly effective strategy to block their production [25]. In the present study, dorsal skin samples from TPA group showed increased levels of the pro-inflammatory cytokines TNF-α, IL-1β, IL-6, and IL-17, which were decreased in mice treated with **MGMG-A** or **MGDG** fraction. On the other hand, IL-10 is considered an anti-inflammatory cytokine since it inhibits T cells and macrophages’ pro-inflammatory cytokine production [26]. In this sense, IL-10 is a rapid-response cytokine that ameliorates the acute immune response that occurs in recurrent diseases such as psoriasis or IBD [27,28]. In this acute experimental epidermal hyperplasia model, dorsal skin reacted to TPA by increasing IL-10 production versus a sham, but treatments interestingly prevented this production. These results are in line with a previous study of murine recurrent colitis in which it is proposed that pre-treatment with an oxylipin-containing lyophilized biomass from microalgae kept levels of pro-inflammatory cytokines low, thus the production of IL-10 was not required, supporting a less hyperactive immune response in the recurrent colitis model [29].

After examining the preventive effects of the compound **MGMG-A** and fraction **MGDG** obtained from *I. galbana*, we aimed to optimize the permeation behavior of these substances with respect to previously acetone-dissolved preparations. In skin diseases such as psoriasis, local topical delivery can be improved by following two main approaches. Firstly, the suitable choice of formulation can optimize the local targeting. Secondly, the physicochemical parameters of the drug itself, such as lipophilicity, can also affect the degree of delivery.

Concerning the choice of carrier composition, different formulations, including hydrophilic gel, cream, and ointment, were prepared and analyzed. For these studies, a fluorescent hydrophobic marker such as rhodamine 6G, with an oil/water partition coefficient of 2.62, was added as a model drug [30]. The in vivo CLSM study defined the cream as the best vehicle to dig deeper into the skin, preserving the entity of different tissue layers. This may be due to the fact that the surfactant-like properties of this heterogeneous disperse system could enhance the amount of permeated rhodamine 6G. On the other hand, a control solution using ethanol as solvent and hydroalcoholic Carbopol^®^ gels also exhibited high drug permeation. This could also be attributed to the presence of ethanol in the formulation, which was used as a solubilizer and permeation enhancer. On the contrary, the deposition of the fluorophore from lipid ointment onto the skin was very low. This would be ascribed to the hydrophobic nature of the vehicle, which, being insoluble, did not penetrate through the skin but remained on the surface, as visualized in Figure 4. Usually, the permeation pathway across the *SC* resides in the intercellular lipid domains. Conversely, at the viable epidermis level, the fluorophore is not only restricted to the cell membranes but also accumulates in the cytosol. In this study, a relationship between the lipophilicity of the applied formulation on the accumulation of the fluorophore in *SC* and viable epidermis was observed. Although the fluorescent label was applied to the *SC* of mice, only low relative accumulation in the deeper layers of the skin was observed for the most lipophilic vehicle, the ointment. This can be explained since the lipid lamellae constitute only a small region of the *SC* compared to the corneocytes. However, the adjacent viable epidermis is much more brightly stained than *SC* in samples from the rhodamine-loaded cream and hydrogel formulations, because in this layer the label can distribute throughout the entire epidermis, which results in a brighter appearance [31].

Once we selected the cream for further studies, an ex vivo permeation process was planned for comparison of **MGDG**-loaded cream with **MGDG** reference solution and Dex-cream. In this assay, the permeation through mice excised skin was determined using a Franz diffusion apparatus and degassed absolute ethanol. Since the goal of this study was to find the experimental conditions that allowed a fast and accurate method to evaluate the permeability properties of different formulations across the skin, we decided to use ethanol as the solvent forming the receiving compartment because the assayed molecules (**MGDG** and Dex) are freely soluble in this medium. This solvent has been used for analyzing the skin diffusion kinetic of other molecules, such as α-tocopherol acetate [32]. However, the use of this solvent has the potential to extract lipids from the *SC* and to artificially increase skin permeability. Since all the formulations tested in these experiments had ethanol as the penetration enhancer interacting with skin constituents to increase drug flux, this would not affect the relative results.

The formulations were detected in the receiver medium in a time-dependent manner and cumulative amount permeated curves were plotted. Concerning **MGDG** cream with respect to the **MGDG** ethanolic solution, the difference in the permeability percentage may be attributed to the surfactant-like properties of emulsion components and their affinity for the phospholipids of the skin membrane [33]. A common mechanism of action of surfactants as penetration enhancers involves firstly a “push” effect to increase the drug solubility and hence to create a high concentration gradient. Secondly, a “pull” effect is related to the flux of the permeation enhancer through the skin, which can induce skin structural transformations [34]. In addition, findings from our study indicate that the cream exerted the best effect in increasing the skin permeation of **MGDG** compared to Dex, in which the permeated amount was lower (15 ± 3.1%). In accordance with other studies [35], this behavior can be attributed to the lower partition coefficient of this molecule (logP 1.83) with respect to **MGDG** [13]. The interaction of the drug between the water and oil phase can determine the extent of lowering of the thermodynamic activity in external phase, which is in contact with the skin. Partitioning of the drug into internal oily phase (higher logP) is due to the hydrophobic characteristic of the drug. As both molecules are highly lipophilic substances, the extremely slow permeation of Dex from the emulsion through the mice skin can be explained by the fact that the diffusion of drug through the oily phase is the limiting step for drug permeation [36].

An important consideration in psoriasis is the skin’s condition. Topical formulations may be applied either to opened lesions that have lost *SC* barrier properties or to thickened lesions that represent an additional barrier to absorption [37]. Thus, our findings suggest that a hydrophilic vehicle (cream) could be an interesting alternative to improved topical delivery of **MGDG** in both conditions.

Based on the results above, we carried out a second TPA-induced hyperplasia model in mice using **MGDG**-cream. In this experimental model, TPA was applied 1 h before the administration of **MGDG**-containing cream to ensure the correct absorption of the product as well as increase its permeation and protect the lipid integrity. Our data showed that dorsal application of TPA caused similar manifestations to those detected in the previous model. Consistent with the anti-inflammatory activity of glycolipids, pre-treatment with **MGDG**-cream ameliorated macroscopic cutaneous lesions, skin edema, and MPO activity induced by repetitive application of TPA, which was correlated with the histological study. Interestingly, the reduction of skin edema and MPO activity was higher with **MGDG**-cream than that detected in the **MGDG** dissolved in acetone, reaching similar levels to those of the reference corticosteroid Dex-cream.

It has been shown that topical application of TPA activates intracellular transduction signals, enhancing aberrant expression of COX-2 in mouse skin [38,39]. To further elucidate the mechanisms for the anti-inflammatory role of **MGDG**-cream in damaged skin, we detected this protein expression by immunohistochemistry. The results evidenced that pre-treatment with **MGDG**-cream markedly attenuated COX-2 expression in TPA-stimulated mouse skin, reaching similar levels to Dex-cream. Furthermore, this effect was confirmed in TNF-α-stimulated HaCaT human keratinocytes pre-treated with different concentrations of **MGDG**, showing that this glycolipid fraction reduced COX-2 levels at 50 µg/mL (*data not shown*). These findings at least partly suggest that suppression of COX-2 expression may be involved in the preventive effect of this glycolipid fraction on TPA-induced epidermal hyperplasia.

## 4. Materials and Methods

### 4.1. Glycolipids

The extraction of dried biomass of *I. galbana* with acetone/methanol and the separation of the extract to obtain the fraction glycolipids has been previously described [8]. The pure compound **MGMG-A** was isolated during the microalgae products separation by C18 SPE cartridges (Supelco, Bellefonte, PA, USA), the elution with MeOH/H_2_O (90:10, *v*/*v*) and the separation by reverse-phase HPLC (Merck, Darmstadt, Germany) using MeOH/H_2_O (98:2, *v*/*v*). The spectroscopic data obtained for this compound, which matched those described in the literature, led to its identification as monogalactosylmonoacylglyceride (2*S*)-1-*O*-[(6*Z*,9*Z*,12*Z*,15*Z*)-octadeca-6,9,12,15-tetraenoyl]-3-*O*-β-d-galactopyranosylglycerol (**MGMG-A**) [40]. On the other hand, the separation of the extract of *I. galbana* previously described led to obtaining a fraction of monogalactosyldiacylglycerides (**MGDG**). In the **MGDG** fraction 10 compounds have been identified and the major **MGDG**s are (2*S*)-1-[(3*Z*,6*Z*,9*Z*,12*Z*,15*Z*)-octadeca-3,6,9,12,15-pentaenoyl]-2-*O*-[(6*Z*,9*Z*,12*Z*,15*Z*)-octadeca-6,9,12,15-tetraenoyl]-*O*-3-*O*-β-d-galactopyranosylglycerol, and (2*S*)-1-*O*-tetradecanoyl-2-*O*-[(6*Z*,9*Z*,12*Z*,15*Z*)-octadeca-6,9,12,15-tetraenoyl]-3-*O*-β-d-galactopyranosylglycerol [9].

### 4.2. Cell Culture

HaCaT human keratinocytes were obtained from the American Type Culture Collection and maintained in high glucose Dulbecco’s modified Eagle’s medium (DMEM, GIBCO, Grand Island, NY, USA) supplemented with 10% fetal bovine serum, 2 mM l-glutamine, 100 U/mL penicillin, and 100 mg/mL streptomycin in an atmosphere of 5% CO_2_ at 37 °C.

### 4.3. Cell Viability Assay

Viability of HaCaT cells upon exposure to glycolipid compounds was determined by the sulforhodamine B (SRB) assay [41]. Briefly, the cells were seeded into 96-well plates at 1 × 10^4^ cells/well. After 24 h, cells were incubated with compounds at the final concentrations range of 10–100 µM or 10–100 μg/mL (100 µL/well) that were prepared by dilution of stock solutions (10 mM) in DMSO in fresh medium. After 24, 48, and 72 h, cells were fixed with 50 μL of trichloroacetic acid (TCA 50% *v*/*v*) at 4 °C for 1 h and processed as described in the literature.

### 4.4. Determination of IL-6 and IL-8 Production

HaCaT cells were seeded in six-well plates (2 mL/well) at 5 × 10^5^ cells/well. After 24 h, the cells were treated with different concentrations of **MGMG-A** (10, 30 and 50 μM) and **MGDG** (10, 30 and 50 μg/mL) and dexamethasone (Dex) (1 μM) for 1 h, and then stimulated with TNF-α (10 ng/mL) for 24 h. Then, supernatant fluids were collected and stored at −80 °C until IL-6 and IL-8 measurements. Controls contained a medium with equivalent amounts of solvent compared to treatments, and were incubated with and without TNF-α. Commercial enzyme-linked immunosorbent assay (ELISA) kits (Diaclone GEN-PROBE, Besançon, France) were used to quantify cytokines according to the manufacturer’s protocol. The absorbance at 450 nm was read by a microplate reader.

### 4.5. Animals

For the present study, eight-week-old female Swiss CD-1 mice (25–30 g) were supplied by Janvier-Labs (Le Genest St. Isle, France). Mice were maintained in our animal laboratory under standard conditions (temperature of 24–25 °C, humidity of 70–75% and 12 h light–12 h dark cycle). Mice were allowed free access to a standard diet (Panlab, Barcelona, Spain) and water ad libitum. Dorsal hair of the mice was removed using electric clippers and depilatory skin cream (Deliplus, Barcelona, Spain) in order to maintain a hair-free skin area to carry out the topical treatments. All studies were performed in accordance with the recommendations of the European Union regarding animal experimentation (Directive of the European Council 2010/63/EU). The experiments followed a protocol approved by the Animal Ethics Committee of the University of Seville.

### 4.6. TPA-Induced Epidermal Hyperplasia Model and Glycolipid Treatments

We evaluated the effect of glycolipids from *I. galbana* on TPA-induced hyperplasia in murine skin. Briefly, dorsal skin of female Swiss mice was shaved and 24 h later, animals that displayed no evidence of hair regrowth or injury were assigned to the different groups.

In a first set of experiments (*n* = 10 per group), **MGMG-A** and **MGDG** dissolved in acetone (10 µg/µL, 200 µg per site) or the reference agent Dex dissolved in acetone (10 µg/µL, 200 µg per site) were topically administered to the shaved dorsal skin of animals in an area of 1 cm^2^ by using a micropipette (total volume, 20 µL). Vehicle (acetone) was administered in a comparable volume to sham and TPA group. After 30 min, TPA (2 nmol per site, dissolved in acetone) was topically applied to the same areas, except the sham, which received a comparable volume of acetone (day 0). This procedure was repeated for two consecutive days [2]. Mice were sacrificed on day 3 by cervical dislocation and punch biopsies from the treated dorsal skin were weighed to evaluate edema before further processing for histology and biochemical parameters.

### 4.7. MPO Activity

Myeloperoxidase (MPO) activity was assayed as a marker of neutrophil infiltration according to the method of Grisham et al. [42]. The tissue was thawed, weighed, and homogenized in 10 volumes of 50 mM-PBS (pH 7.4). The homogenate was centrifuged at 20,000× *g* for 20 min at 4 °C. The pellet was again homogenized in 10 volumes of 50 mM-PBS (pH 6) containing hexadecyl trimethylammonium bromide (0.5%) and 10 mM-EDTA. This homogenate was subjected to one cycle of freezing/thawing and a brief period of sonication. A sample of the homogenate (50 µL) was added to a 96-well microplate and incubated at 37 °C for 3 min with a mixture containing *o*-dianisidine dihydrochloride (0.067%), hexadecyl trimethyl-ammonium bromide (0.5%) and 0.3 mM-H_2_O_2_. Changes in absorbance at 450 nm were measured with a microplate reader (Labysystem Multiskan EX, Thermo Scientific, New York, NY, USA). One unit of MPO activity was defined as the amount of enzyme present that produced a change in absorbance of 1.0 unit/min at 37 °C in the final reaction volume containing the acetate. Results are expressed as units/mg tissue.

### 4.8. Histological Study

Tissue samples from the dorsal skin of four animals were fixed in 4% buffered paraformaldehyde, dehydrated by increasing concentrations of ethanol and embedded in paraffin. Tissue sections cut to 7 μm on a rotary microtome (Leica Microsystems, Wetzlar, Germany) were mounted on slides, deparaffinized with xylene, rehydrated through graded alcohols, and stained with hematoxylin and eosin (H&E) according to standard protocols. The tissues were analyzed by a blinded observer under an Olympus BH-2 microscope (GMI Inc., Ramsey, MN, USA) for determination of histopathological changes. Epidermal thickness was measured using Scientific Imaging Systems (Biophotonics ImageJ Analysis Software; National Institutes of Health, Rockville, MD, USA).

### 4.9. Measurement of Cytokines in Skin Homogenates

Frozen skin biopsies were homogenized in three volumes of ice-cold tissue lysis buffer containing PBS (pH 7.2) with 0.1 M of EDTA, 1 mg/mL of leupeptin, 1 mg/mL of pepstatin, 1 mg/mL of aprotinin, and 1 mM phenylmethylsulfonyl fluoride. Homogenates were centrifuged at 12,000× *g* for 10 min at 4 °C. Supernatant fluids were stored at −80 °C until measurements. Levels of TNF-α, IL-1β, IL-6, IL-17 and IL-10 were measured by quantitative Enzyme-Linked ImmunoSorbent Assay kits (ELISA) (Peprotech, Hamburg, Germany), according to the manufacturer’s instructions. Data are reported as pg/mg tissue.

### 4.10. Skin Topical Formulations

Hydrophilic gel. Carbopol^®^ 934P (Lubrizol, Cleveland, OH, USA) was selected as a gelling agent at 1% (*w*/*v*) due to its widespread use in pharmaceutical formulations and fast dispersion in water. Rhodamine 6G solution in ethanol absolute (10 µg/µL) was gradually added to the polymer dispersion under magnetic stirring. The dispersion was neutralized with triethanolamine to obtain an adequate consistency suitable for topical application.

Cream. This O/W emulsion was prepared by gradually adding rhodamine (10 µg/µL), **MGDG** (10 µg/µL) or Dex (10 µg/µL) solution in ethanol absolute to a cold mix excipient composed of Caprylic/Capric Triglycerides, Glycol Stearate, PEG-3 Glyceryl Cocoate and Steareth-7 previously heated to improve the drug interposition. The final formulations contained 0.2% (*w*/*w*) **MGDG**.

Lipophilic ointment. This formulation was obtained using melt emulsification combined with stirring. Briefly, Brij^®^ 72 (5% *w*/*w*) and white soft paraffin (77.3% *w*/*w*) were blended under gentle stirring in a water bath at 70 °C to form the lipid phase. Successively, liquid paraffin (5% *w*/*w*) and α-tocopherol (0.002% *w*/*w*) were added into the lipid phase until complete interposition at 60 °C. Then, rhodamine dissolved in propylene glycol (10 µg/µL) was added to the mixture. Meanwhile, two solutions of EDTA (0.0065% *w*/*w*) and disodium phosphate dihydrate (0.026% *w*/*w*) were prepared with distilled water at 60 °C and added dropwise to the lipid phase, with moderate magnetic stirring at 150 rpm for 20 min. Finally, the rhodamine-loaded ointment was maintained at room temperature for further use.

### 4.11. Permeation Studies

In vivo skin depth permeation by confocal laser scanning microscopy (CLSM). Confocal studies were performed in order to investigate the penetration ability of several formulations through the different skin layers [43]. Towards this aim, Carbopol^®^ hydrogel, cream, and ointment were prepared by adding a hydrophobic fluorescent probe, i.e., rhodamine 6G (100 mg per site, equivalent at 200 µg of compound dissolved in ethanol), in the lipid or in the water-ethanol phase, according to the composition of formulations. Rhodamine 6G was selected as an equivalent marker for **MGDG** or Dex due to their comparable lipophilic properties. Appropriate samples of these formulations were taken and placed on *SC* of mice and maintained in contact with the skin for 24 h. At the end of the experiment, the remaining preparation was carefully washed with purified water from the skin surface. Then, dorsal skin was excised and rinsed with pH 7.4 phosphate buffer solution, rapidly frozen in liquid nitrogen and then stored at −80 °C. Sections of skin (50 µm thickness) were then perpendicularly cut with a cryomicrotome and examined to investigate the fluorescent marker distribution in the different skin layers. Analysis was carried out using a Leica TCS SP II CLSM (Leica, Heidelberg, Germany) equipped with a Kr-Ar-He-Ne ion laser and a Leica DM IRE 2 microscope endowed with HCPL Fluotar Leica 10× and 20× dry objectives and HCXPLAN APO Leica X40 multi-immersion objective (numeric aperture 0.85). For excitation of the fluorescent label the 488 nm wavelength was used and the fluorescence emission was detected at 520 nm.

From the confocal images, a mathematical treatment was carried out in order to evaluate the intensity of fluorescence in the samples, as appeared in the histogram curve provided from Leica software. Statistical calculations were as follows:

(1) $$\mathrm{Arithmetical}\;\mathrm{mean}\;\mathrm{value}:\;\mu \left(I\right)=\frac{1}{N_{\mathrm{pixel}}}\times \sum^{\text{​}}I_{i},$$

(2) $$\mathrm{Average}\;\mathrm{image}\;\mathrm{energy}:\;I_{\mathrm{mean}}^{2}=\frac{1}{N_{\mathrm{pixel}}}\times \underset{\mathrm{pixel}}{\sum }I_{i}^{2},$$

(3) $$\mathrm{Root}\;\mathrm{mean}\;\mathrm{square}\;\mathrm{value}:\;I_{\mathrm{mean}}^{2}=\sqrt{\frac{1}{N_{\mathrm{pixel}}}\underset{\mathrm{pixel}}{\sum }I_{i}^{2}},$$

(4) $$\mathrm{Skewness}\;\mathrm{of}\;\mathrm{the}\;\mathrm{distribution}:\;\frac{1}{N}\underset{i}{\sum }{\left[\frac{I_{i}-\mu \left(I\right)}{\sqrt{VAR\left(I\right)}}\right]}^{3},$$

where *I* is the energy intensity, *I_i_* is the energy intensity of each pixel, *I*_mean_ is the mean value of energy intensity, *N*_pixel_ is the number of pixels of the image, µ(*I*) is the arithmetical mean of the energy intensity and *VAR* is the variance.

Ex vivo permeation studies. Diffusion studies were carried out using a Franz diffusion cell apparatus (SES-Gmgh Analyses system, Bechenheim, Germany) with an effective diffusional area of 3.14 cm^2^. Excised mice skin was used as a membrane. Animals were sacrificed and full thickness dorsal skin was excised. A specific portion of the skin was cut and used for the permeation study after washing it with distilled water. The study was carried out following the methodology previously reported [44]. Animal skin was inserted between the donor and receiving compartments and adjusted by means of a pinch clamp. The receiving chamber was filled with 14.5 mL of degassed ethanol absolute and thermostated by means of a water bath circulator and a jacket surrounding the cell, maintaining 32 °C in the skin surface. The receiving medium was continuously stirred to avoid the diffusion layer effect.

Once we selected the cream carrier from the previous study, **MGDG**-cream (0.2% *w*/*w*), **MGDG** control solution (0.2% *w*/*w*), and Dex-cream (0.2% *w*/*w*) were accurately measured and placed on *SC* in the donor compartment and sealed with parafilm. Aliquots of 0.5 mL were withdrawn from the receiving medium at predetermined time intervals (0, 0.5, 1, 2, 3, 4, 5, 6 and 24 h) according to international guidelines and the same volume was replaced with fresh ethanol absolute at the same temperature. These samples were quantified by HPLC (Hitachi Elite LaChrom, Barcelona, Spain) for **MGDG** quantification and spectrophotometry UV-visible (Agilent 8453, Barcelona, Spain) was used for Dex quantification. HPLC system is equipped with an L-2130 isocratic pump, a diode array detector L-2455 and L-2200 autosampler. The chromatographic separation was performed on a reverse-phase LichroCART^®^ C18 column (5 μm, 4.6 mm ID × 150 mm, Agilent, Santa Clara, CA, USA) using as mobile phase acetonitrile (ACN, solution A): formic acid solution ((0.1% *v*/*v*), solution B) adjusted to pH 2.67 in the following gradient (*v*/*v*): 0–6 min, 90% A, 10% B, flux 1 mL/min; 6–15 min, 100% A. The injection volume was 30 µL. The cumulative amount of drug in receptor chamber for the three formulations (**MGDG**-cream, **MGDG** control solution and Dex control solution) was plotted as a function of time (*t*, h). The cumulative amount (%) of drug permeated through the skin (*P*%) was determined as per the following equation [45]:

(5) $$P\%=\frac{C_{n}\times V+\sum_{i=1}^{n-1}C_{i}\times V_{i}}{M}\times 100,$$

where *C_n_* is the drug concentration of the *n*th sampling point (mg/mL), *C_i_* is the drug concentration of the *i*th sample point (mg/mL), *V* is the total volume (14.5 mL) of liquid in receiving pool, *V_i_* is the volume (0.5 mL) of the *i*th sampling points and *M* is the mass of drug (**MGDG** or Dex).

### 4.12. TPA-Induced Epidermal Hyperplasia Model and **MGDG**-Cream Treatment

Given acetone is considered moderately toxic and irritant and we aim to elaborate an optimal glycolipid-containing pharmacological formulation, we evaluated the effect of **MGDG**-cream on TPA-induced hyperplasia in murine skin. The dorsal skin of female Swiss mice was shaved as described above.

In this second experimental model (*n* = 10 per group), a pre-treatment was carried out two days (day −2 and day −1) before the first TPA challenge (day 0). **MGDG**-cream formulation (100 mg per site, containing 200 µg of **MGDG** dissolved in ethanol at 10 µg/µL), Dex-cream (100 mg per site, equivalent at 200 µg of compound dissolved in ethanol at 10 µg/µL) or vehicle (cream with a comparable volume of ethanol) were applied to the shaved dorsal skin of the animals in an area of 1 cm^2^ using a syringe. On day 0, TPA (2 nmol per site, dissolved in ethanol) was topically applied to the same areas using a micropipette (total volume of 20 µL). After 1 h, **MGDG**-cream, Dex-cream, or vehicle was topically administered. Mice were anesthetized with ketamine (100 mg/kg of animal) and diazepam (5 mg/kg of animal) during treatments and TPA challenge. This protocol was repeated for two consecutive days. Mice were sacrificed on day 3 by cervical dislocation and punch biopsies from the treated dorsal skin were weighed to evaluate edema, before further processing for MPO activity and histology.

### 4.13. Immunohistochemical Analysis

Staining of COX-2 was performed using a streptavidin-biotin-peroxidase method [46]. Paraffin-embedded dorsal skin sections (7 µm) were mounted on slides, deparaffinized with xylene, and rehydrated through graded alcohols. These sections were boiled (10 mM citrate buffer, pH 6.0 for 3 min) for antigen retrieval, followed by cooling at room temperature for 20 min. Endogenous peroxidase was quenched with 0.3% (*v*/*v*) hydrogen peroxide for 20 min. Sections were rinsed with PBS for 10 min. Nonspecific adsorption was minimized by incubating sections in normal horse serum (Vectastain Kit; Vector Laboratories, Burlingame, CA, USA) for 20 min. Subsequently, slides were incubated with rabbit polyclonal anti-COX-2 antibody (Cayman Chemical, Ann Arbor, MI, USA) (1:300) overnight at 4 °C. Then, slides were treated with anti-mouse IgG antibody for 30 min and incubated with the streptavidin–peroxidase complex for 30 min at room temperature (Vectastain Kit; Vector Laboratories, CA, USA). The enzymatic activities were developed with 3,3′-diaminobenzidine (DAB), and the sections were counterstained with hematoxylin. Negative control sections were treated in the same way, omitting the primary antibody [47]. COX-2 immunoreactivity was examined on all sections using a microscope Olympus BX61 (Olympus Optical Co. Ltd., Tokyo, Japan). The quantification of immunohistochemical data was done by counting the number of immunostained brown cells as the percent of total epidermal cells from 10 microscopic fields of immunostained tissues per animal.

### 4.14. Statistical Analysis

All values in the figures and text are expressed as arithmetic means ± SEM. Data were evaluated with GraphPad Prism version 5.00 software (GraphPad Software, Inc., San Diego, CA, USA). In all cases, the Shapiro–Wilk test was used to verify the normality of the data. The Mann–Whitney U-test was chosen for non-parametric values. The parametric values groups were analyzed by one-way analysis of variance (ANOVA) followed by Bonferroni’s Multiple Comparison Test. *p* values < 0.05 were considered statistically significant. In the histological experiment, results shown are representative of at least four independent experiments performed on different days.

## 5. Conclusions

In conclusion, our study demonstrates for the first time the preventive effects of topical administration of the glycolipid **MGMG-A** or a fraction **MGDG** from *I. galbana*, in the inflammatory model of TPA-induced skin hyperplasia. These actions may be associated with a reduction of edema, leukocyte infiltration, pro-inflammatory cytokines production and COX-2 expression in skin mouse. Topical application of **MGDG**-cream enhanced the sample permeability and consequently, increased the preventive effects of this product. Future studies are needed to expand the vision of the mechanisms by which these lipid products improve skin inflammation and will support their potential use in the development of effective therapeutic strategies for skin pathologies as psoriasis.

## Supplementary Materials

The following are available online at www.mdpi.com/1660-3397/16/1/2/s1, Table S1: Viability of HaCaT human keratinocytes treated with different concentrations of **MGMG-A** and **MGDG** fraction isolated from the microalgae *Isochrysis galbana*.

## Abbreviations

CLSM	confocal laser scanning microscopy
Dex	dexamethasone
IL	Interleukin
*I. galbana*	*Isochrysis galbana*
**MGDG**	Monogalactosyldiacylglycerol fraction
**MGMG-A**	Monogalactosylmonoacylglyceride (2*S*)-1-*O*-[(6*Z*,9*Z*,12*Z*,15*Z*)-octadeca-6,9,12,15-tetraenoyl]-3-*O*-β-d-galactopyranosylglycerol
MPO	myeloperoxidase
RMS	root mean square
*SC*	*stratum corneum*
TNF-α	Tumor Necrosis Factor alpha
TPA	12-*O*-tetradecanoylphorbol-13-acetate
