# Supplementary material for: Topical Application of Glycolipids from Isochrysis galbana Prevents Epidermal Hyperplasia in Mice
Source: Mar Drugs. 2017 Dec 25;16(1):2. doi: 10.3390/md16010002 (PMC5793050; doi:10.3390/md16010002)
Supplement: Supplementary file 1 [file marinedrugs-16-00002-s001.pdf]

Supplementary Table 1. Viability of HaCaT human keratinocytes treated with different concentrations of **MGMG-A** and **MGDG** fraction isolated from the microalgae *Isochrysis galbana*. Values are mean ± ES (%) of three independent experiments (N = 3).

	% Viability HaCaT keratinocytes (24 h)				
	5	10	30	50	100
**MGMG-A** (μM)	97.56 ± 2.4	98.6 ± 3.1	97.7 ± 0.9	102.5 ± 2.3	100.3 ± 2.8
**MGDG** (μg/mL)	102.1 ± 2.6	99.6 ± 2.3	102.3 ± 1.5	100.3 ± 3.7	99.5 ± 1.9
